# Supplementary material for: Stacked regressions and structured variance partitioning for interpretable brain maps
Source: Neuroimage. Author manuscript; Available in PMC 2025 May 28. (PMC12117960; doi:10.1016/j.neuroimage.2024.120772)
Supplement: supplementary [file NIHMS2076885-supplement-supplementary.pdf]

# Supplementary Materials — Stacked regressions and structured variance partitioning for interpretable brain maps

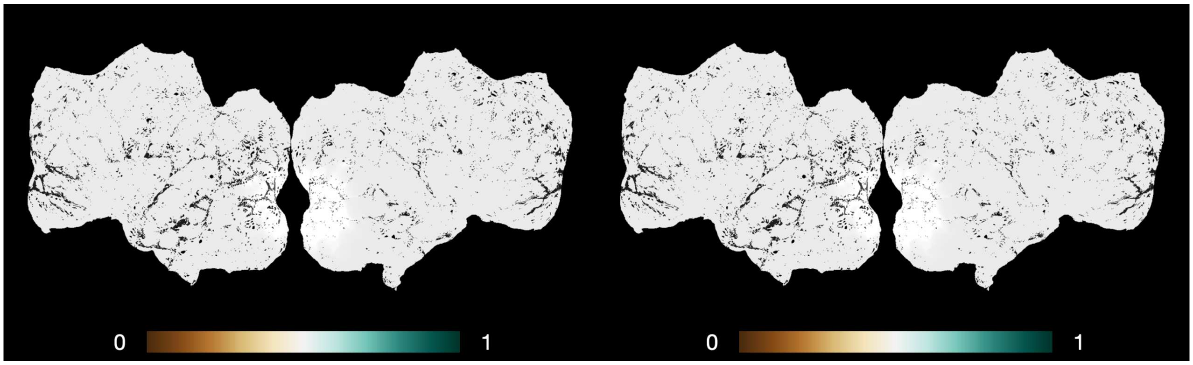


Supplementary Figure S1: Stacking weights for two identical feature spaces shown on a participant's flattened surface. The left map shows the weights for the first feature space and the right map shows the weights for the second feature space. We observe that the two figures are exactly the same, and every weight is 0.5. This result shows that our stacking method gives identical feature spaces the same weight. (Black pixels in both figures are missing voxels in the brain map.)

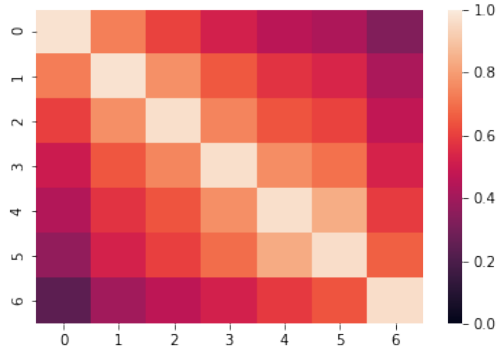


Supplementary Figure S2: Similarity between the different layers of AlexNet, computed over the NSD images.

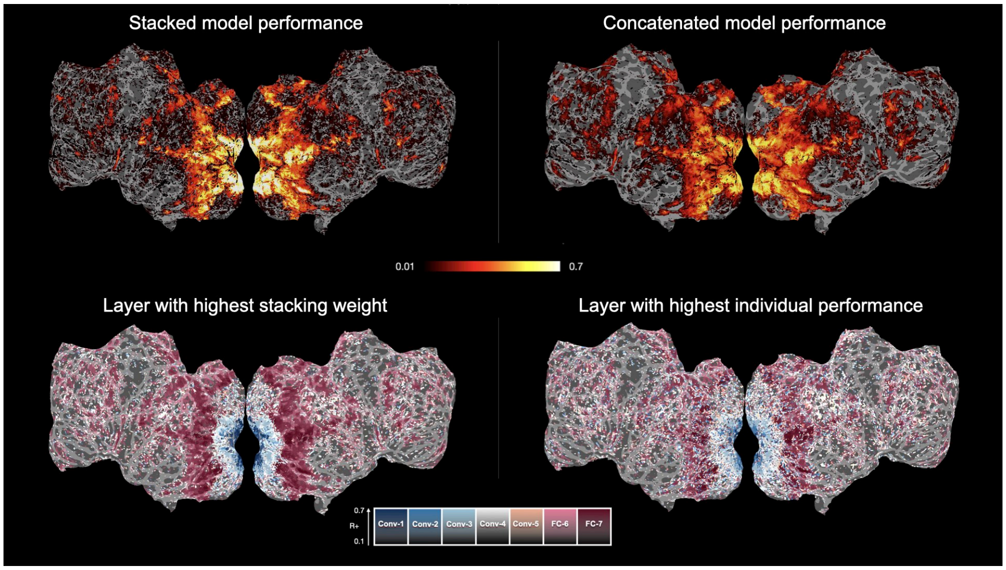


Supplementary Figure S3: [Top] Stacking and concatenation prediction performance for subject S1. [Bottom] Feature attribution maps using the stacking weights (Left) and the layer with maximum performance (Right).

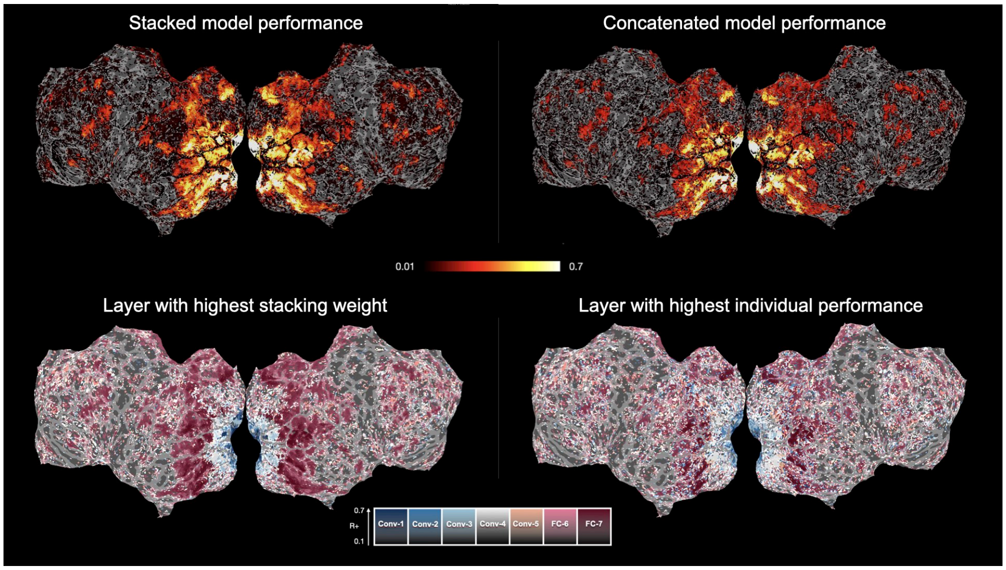


Supplementary Figure S4: [Top] Stacking and concatenation prediction performance for subject S2. [Bottom] Feature attribution maps using the stacking weights (Left) and the layer with maximum performance (Right).

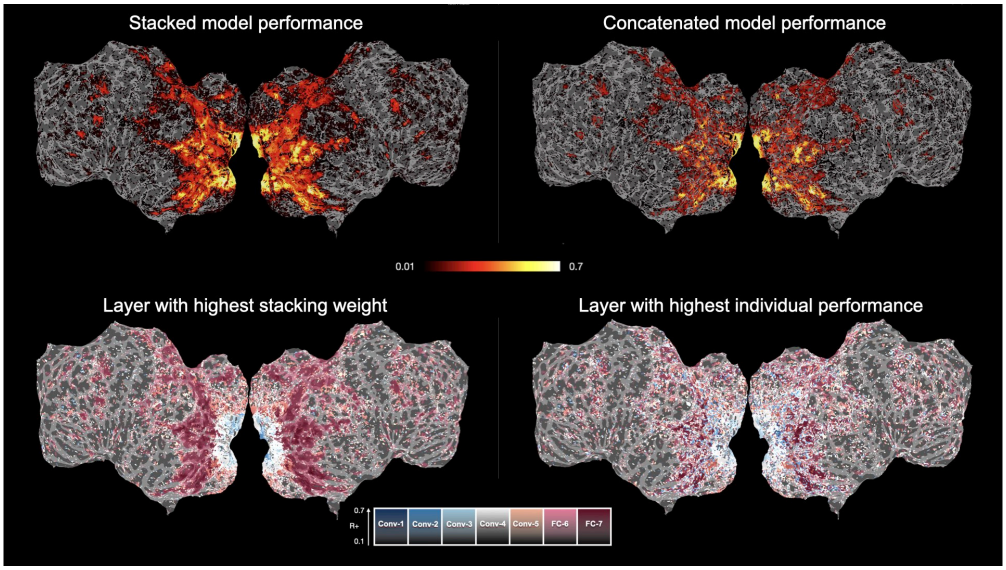


Supplementary Figure S5: [Top] Stacking and concatenation prediction performance for subject S3. [Bottom] Feature attribution maps using the stacking weights (Left) and the layer with maximum performance (Right).

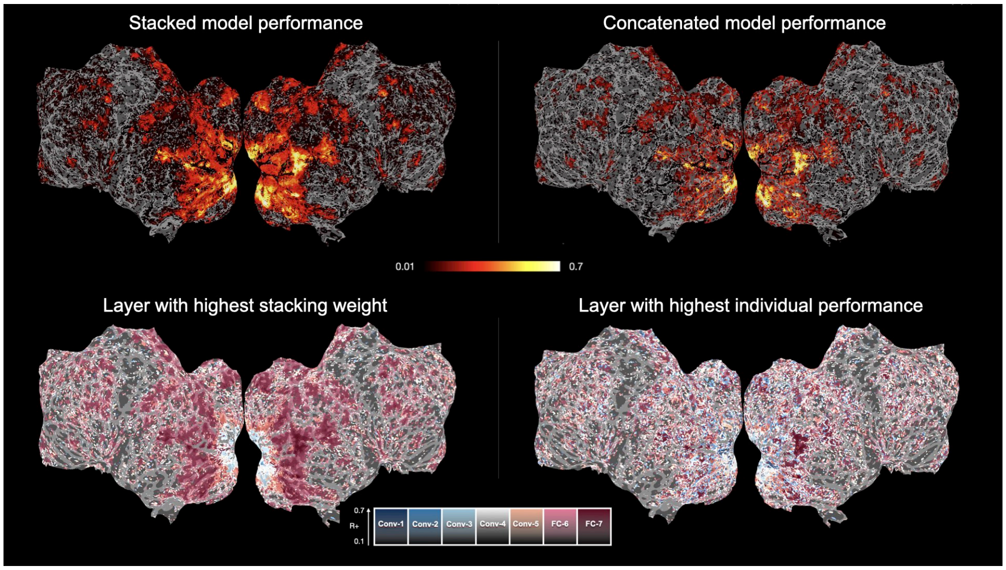


Supplementary Figure S6: [Top] Stacking and concatenation prediction performance for subject S4. [Bottom] Feature attribution maps using the stacking weights (Left) and the layer with maximum performance (Right).

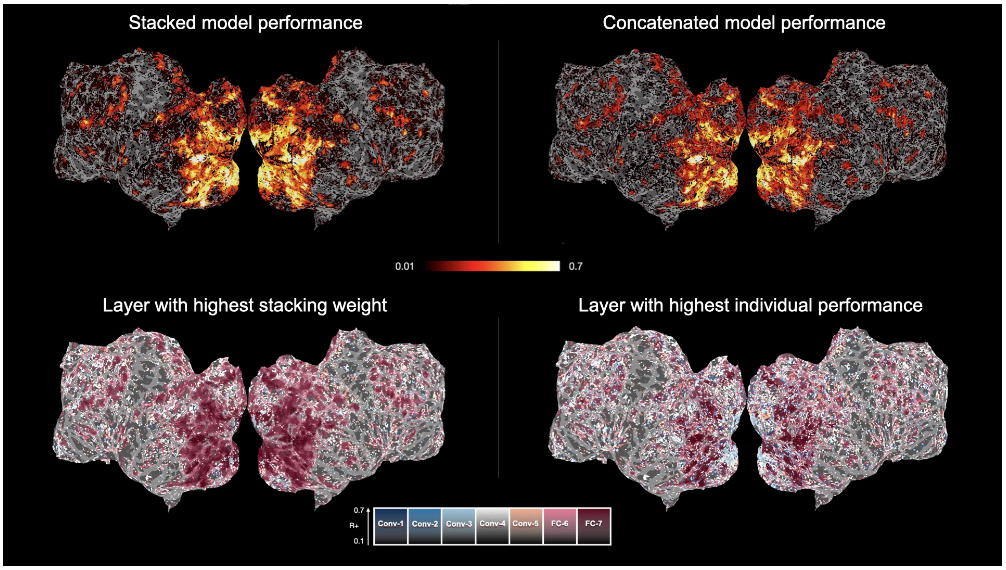


Supplementary Figure S7: [Top] Stacking and concatenation prediction performance for subject S5. [Bottom] Feature attribution maps using the stacking weights (Left) and the layer with maximum performance (Right).

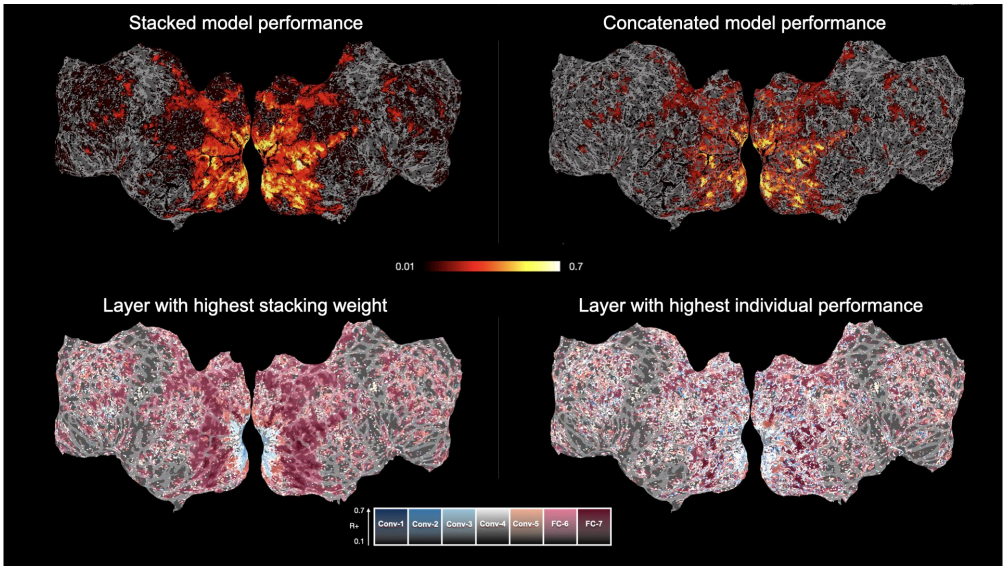


Supplementary Figure S8: [Top] Stacking and concatenation prediction performance for subject S6. [Bottom] Feature attribution maps using the stacking weights (Left) and the layer with maximum performance (Right).

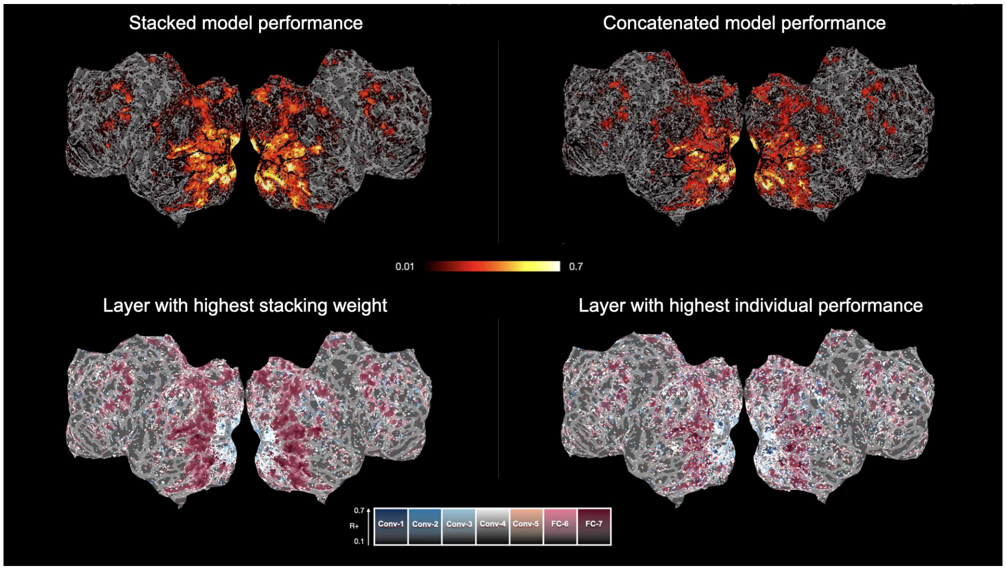


Supplementary Figure S9: [Top] Stacking and concatenation prediction performance for subject S7. [Bottom] Feature attribution maps using the stacking weights (Left) and the layer with maximum performance (Right).

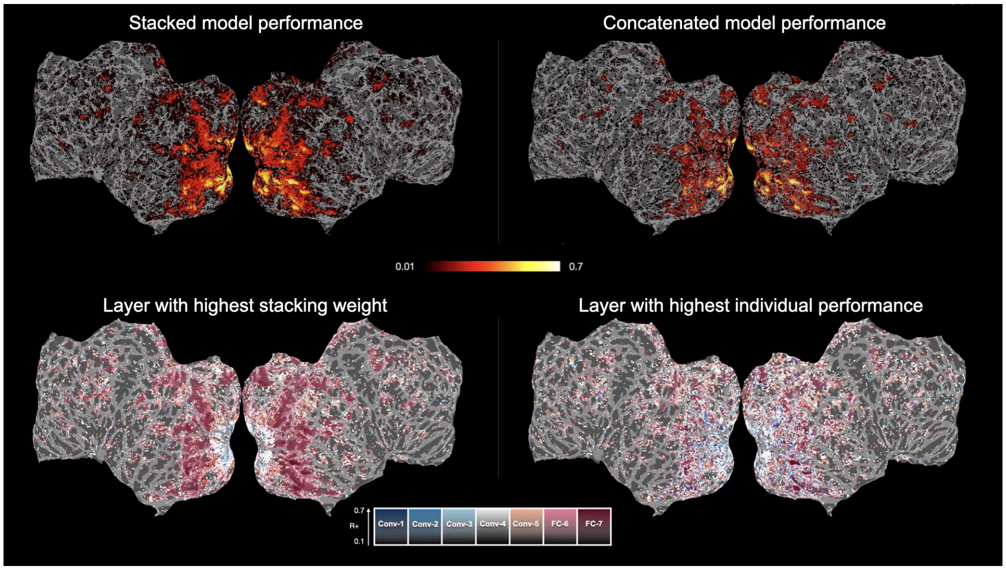


Supplementary Figure S10: [Top] Stacking and concatenation prediction performance for subject S8. [Bottom] Feature attribution maps using the stacking weights (Left) and the layer with maximum performance (Right).

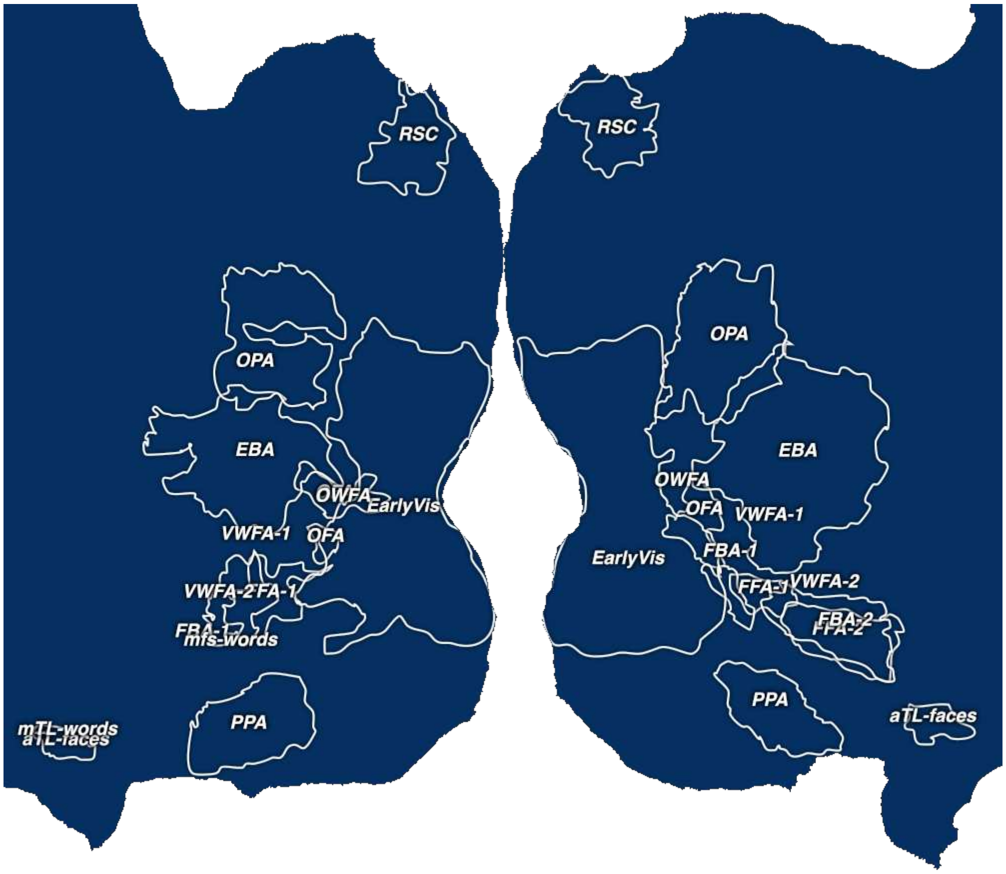


Supplementary Figure S11: Flatmap representation of functional ROIs available in the NSD dataset for subject S1, obtained using the fLoc localizer [72].

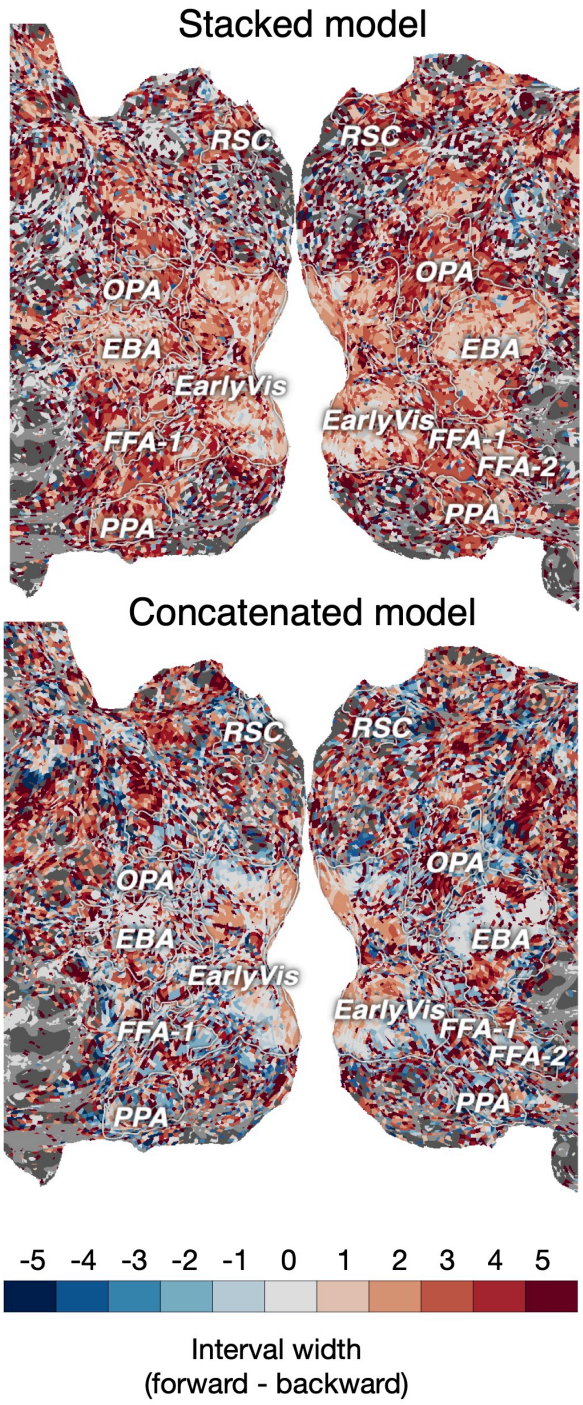


Supplementary Figure S12: Cropped flatmap representation of the width of the interval between the layer obtained using the forward direction and the backward direction of structured variance partitioning, for subject S1.

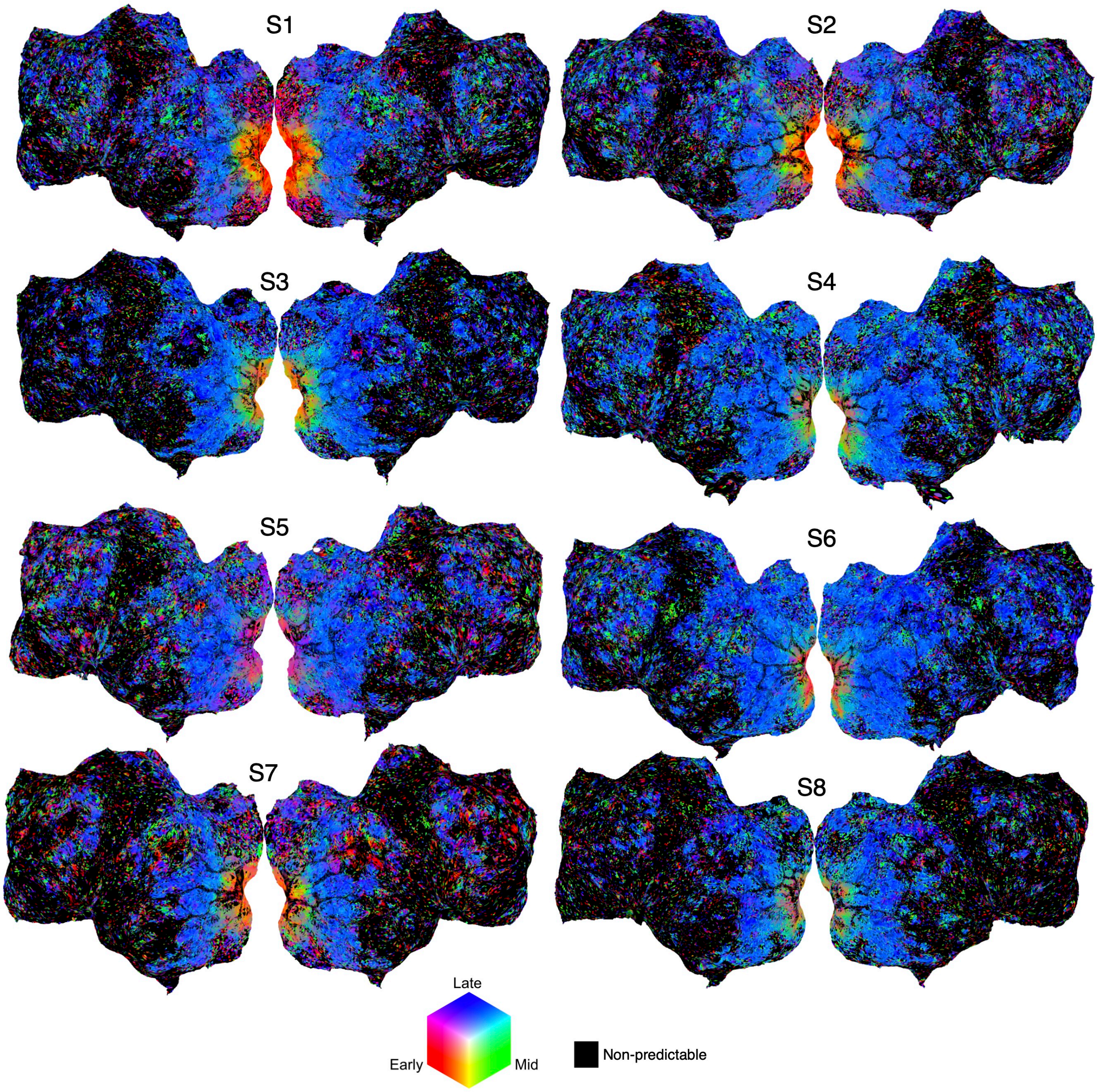


Supplementary Figure S13: We produced a simultaneous visualization of the stacking weights of the AlexNet layers by grouping the layers into three groups: early (1-3), middle (4-5) and late (6-7) layers. We sum the weights of the layers in each group and use different channels to plot them on the flatmap of each subject (early = Red, Middle = Green, Late = Blue). This visualization shows that indeed the change in the layer preference happens continuously, with very few intermediate region having preference for both early and middle or both middle and late layers, and no regions preferring both early and late layers.

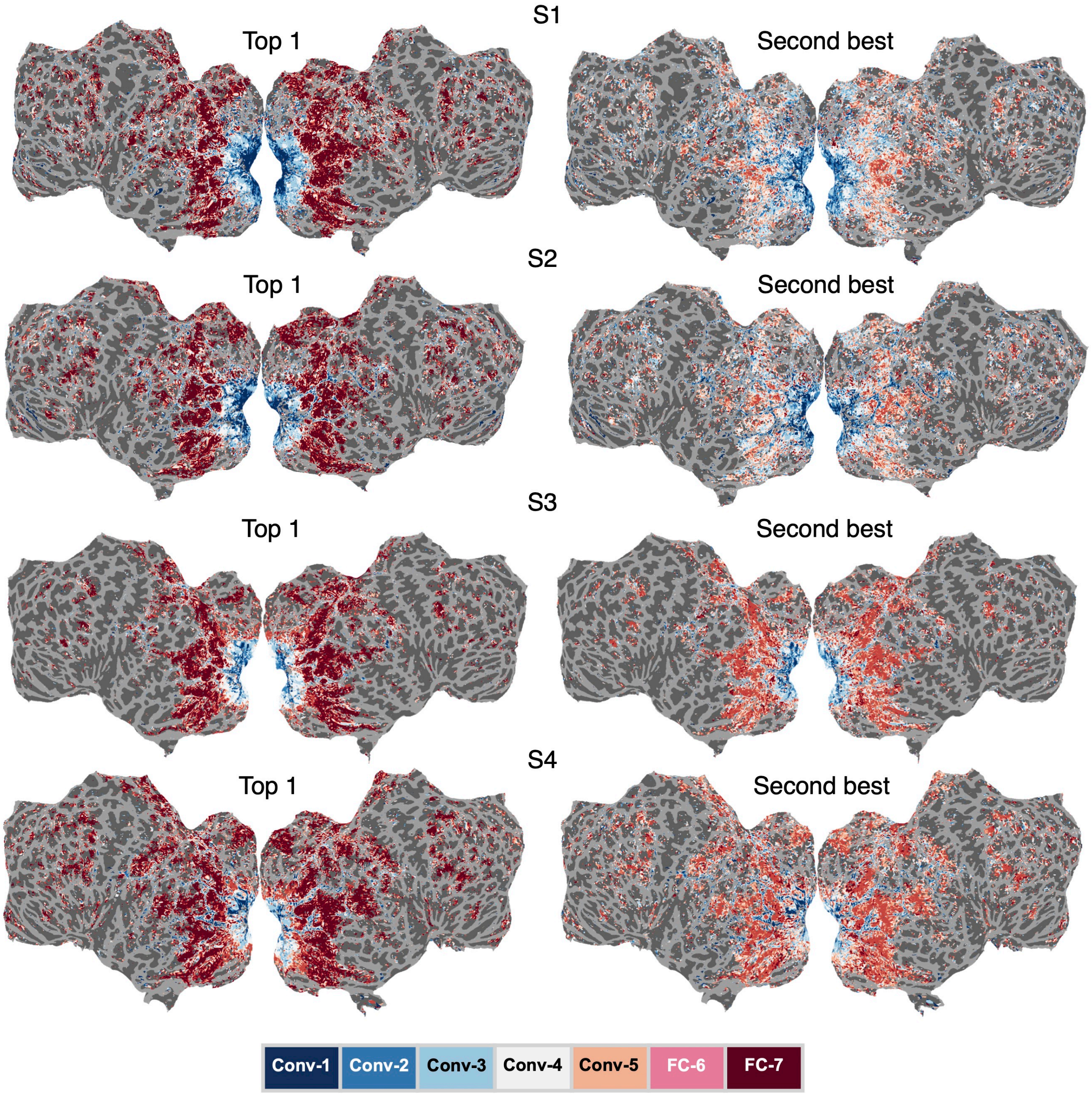


Supplementary Figure S14: We visualize the layer with the second highest stacking weight for each voxel, and juxtapose it to the visualization of the top layer for subjects 1-4. The second best map is close in color to the top 1 layer, meaning that for most voxels, the second best layer is a layer adjacent to the top 1 layer.

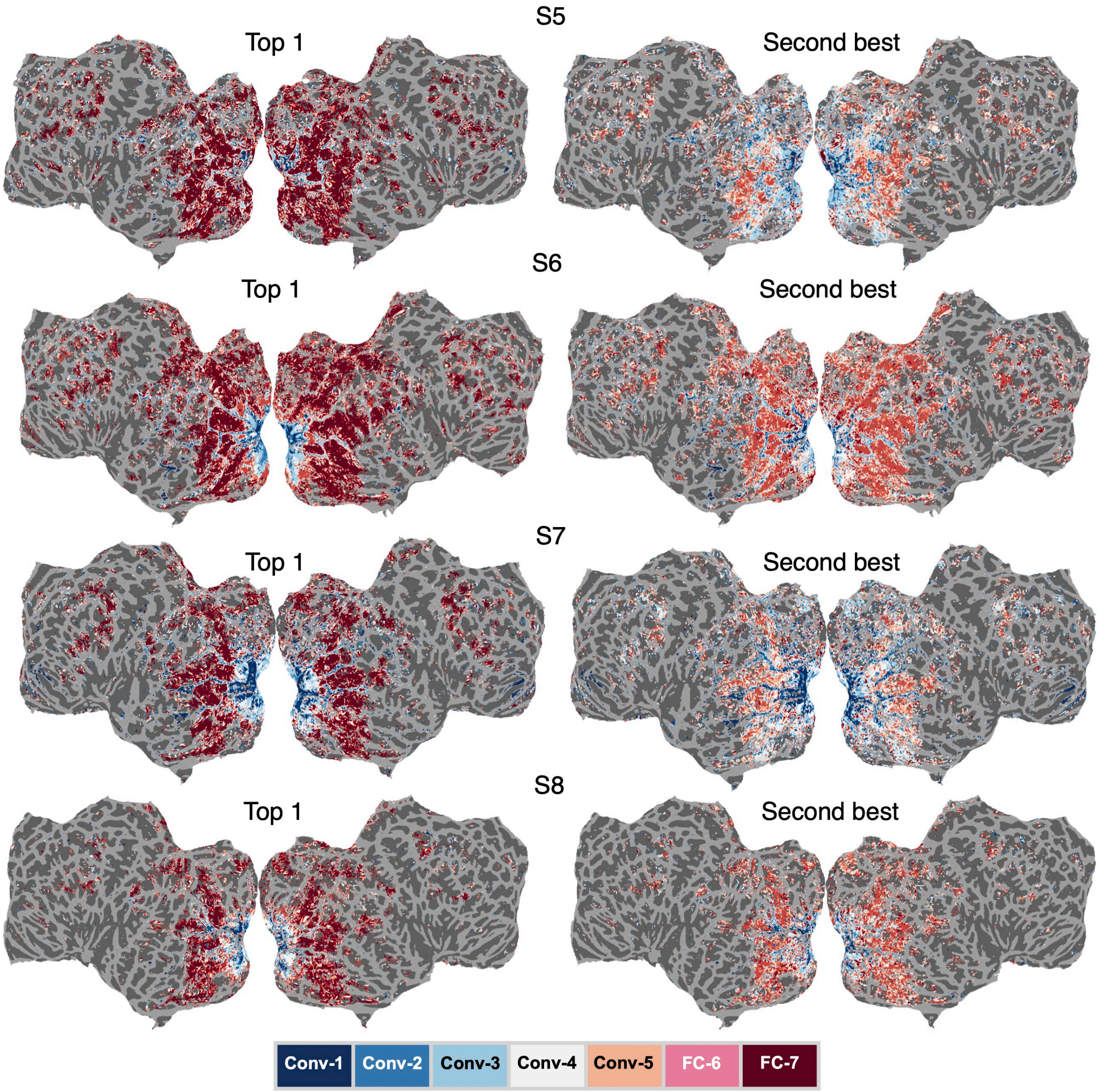


Supplementary Figure S15: We visualize the layer with the second highest stacking weight for each voxel, and juxtapose it to the visualization of the top layer for subjects 5-8. The second best map is close in color to the top 1 layer, meaning that for most voxels, the second best layer is a layer adjacent to the top 1 layer.

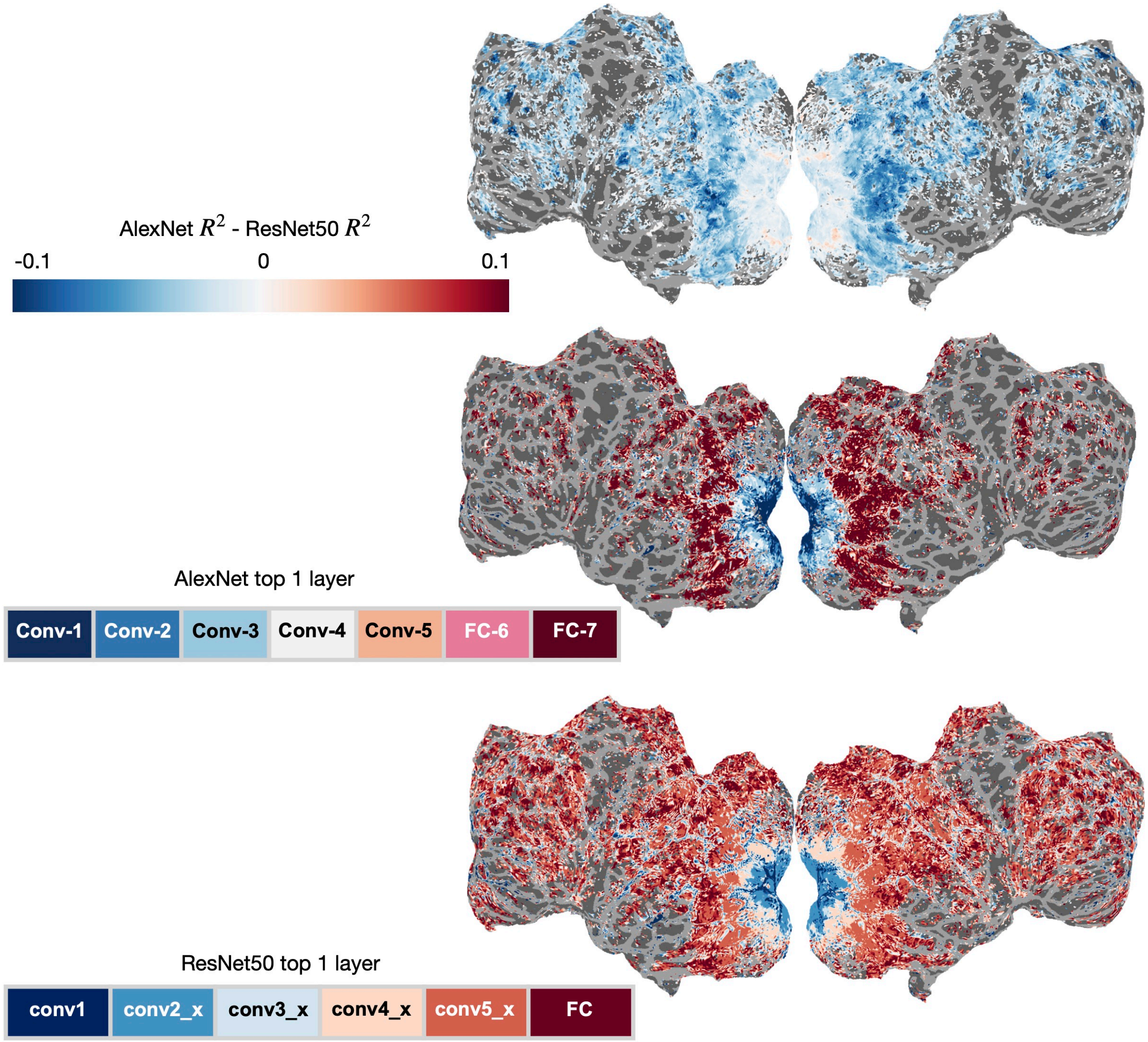


Supplementary Figure S16: Comparison to using features from a pretrained [73] ResNet-50 [53]. We create six feature spaces from the layers of ResNet-50: `conv1` (first convolutional layer), the outputs of the convolutional blocks `conv2_x` to `conv5_x` and `fc` (the final fully connected layer). We find that stacked ResNet-50 performance is similar to staked AlexNet performance in the early visual cortex, with some slight improvement for AlexNet over ResNet-50 in the brain region corresponding to the most peripheral part of the visual field. In high-level visual cortex, we see ResNet-50 far outperforming AlexNet. When looking at the layer-attribution results, the results from ResNet-50 resemble the pattern of the AlexNet results to a considerable extent, with the early visual regions preferring the early layers and the late regions preferring the later layers.

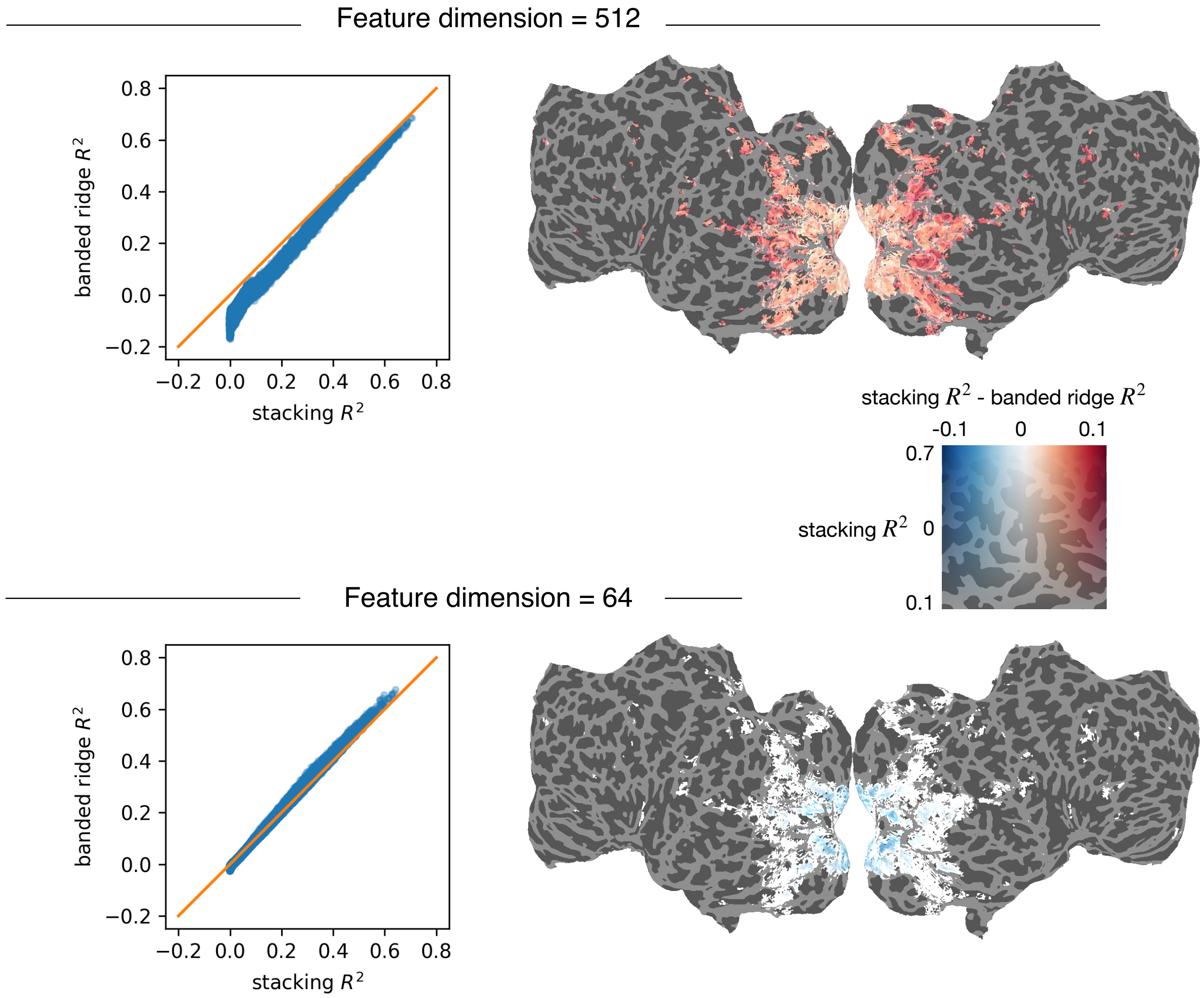


Supplementary Figure S17: Comparison between the prediction performance when using our stacking procedure or the banded ridge procedure [21, 22] with the same input features. [Top] The 512 top PCs were retained for all layers of AlexNet. The stacking performance shows a slight improvement over the banded ridge performance, more pronounced for less predictable voxels. [Bottom] The 64 top PCs were retained for all layers of AlexNet. The banded ridge performance shows a slight improvement over the stacking performance, more pronounced for highly-predictable voxels. These results suggest that banded ridge (which concatenates all the feature spaces together and chooses different penalty parameters for each) is performs well for smaller feature sizes and has a slight disadvantage for larger feature sizes.

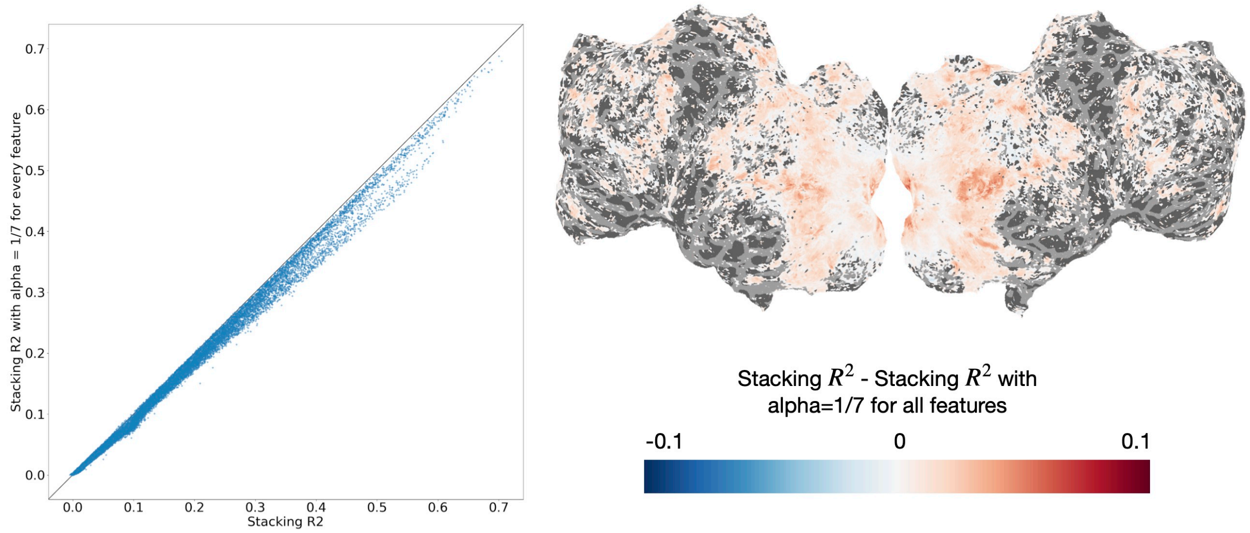


Supplementary Figure S18: Comparison between the prediction performance of our stacking procedure or when fixing the weights of each predictor to be equal (effectively averaging the first level predictors). The stacking performance is uniformly greater or as good as averaging the first level predictors. The advantage of stacking is most pronounced in a large set of the good voxels. Note: the cross-validation step in the first layer results in high penalty when the performance is bad, meaning that the well performing layers in a voxel will have predictions that have higher magnitude than the less well performing layers, and thus will have more weight in the averaging, which might explain why the performance during averaging is close to the stacking performance in many voxels. However, in the good voxels, there is still a considerable improvement, indicating that averaging doesn't suffice.
